# Assumption of the Myths of Romantic Love: Its Relationship With Sex, Type of Sex-Affective Relationship, and Sexual Orientation

**DOI:** 10.3389/fsoc.2021.621646

**Published:** 2021-05-20

**Authors:** Jenny Cubells-Serra, Alejandro Sánchez-Sicilia, Priscila Astudillo-Mendoza, Neli Escandón-Nagel, María José Baeza-Rivera

**Affiliations:** ^1^Departamento de Psicología Social, Facultad de Psicología, Universidad Autónoma de Barcelona, Barcelona, Spain; ^2^Departamento de Psicología, Facultad de Ciencias de la Salud, Universidad Católica de Temuco, Temuco, Chile

**Keywords:** bisexual, consensual non-monogamy, monogamy, polyamory, exclusivity, better-half

## Abstract

Romantic love promotes and lays the foundation for the development of hegemonic affective sex relationships, guiding the normative ways of feeling and experiencing love. This way of conceiving love is an intrinsic part of women's subordination, and it entails a greater tolerance for situations of violence in sex-affective relationships in which the exercise of asymmetric power relations between men and women is legitimized. With the current advent of the postmodern stage, a wide variety of dissident (non-heterosexual) sexual orientations with heterosexual hegemony have been given greater visibility and legitimacy, and new ways of relating to sex affectively have emerged initially opposed to traditional romantic discourse, the fundamental pillar of monogamy. The aim of the present work was to study whether these different ways of linking us and understanding affective sex relations marked a significant difference with respect to the heterosexual monogamous hegemonic model in the assumption of the mythified ideas of romantic love. Therefore, we studied the relationship between sex, sexual orientation, and the type of sex-affective relationship (monogamous or non-monogamous by consensus) in the assumption of the myths of romantic love. For this purpose, an instrument that showed appropriate psychometric properties was created, and a cross-sectional study was carried out with a sample of 1,235 people who completed a self-administered online questionnaire. The results indicated that there were no significant differences according to sex, but there were differences in sexual orientation and type of relationship. It may be concluded that a person, regardless of sex, heterosexual or homosexual, monogamous or who has never had affective sex relations, will have a significantly greater probability of assuming the myths of romantic love than a person with a sexual orientation other than heterosexual or homosexual and who is in a non-monogamous consensual relationship.

## Introduction

Affective sexual relationship is a phenomenon affected by transitional events in the middle of a postmodern stage were originally opposed discourses and ways of understanding these types of relationships coexist and conflict between each other. In this context is produced a multiphrenic state, in which people start to experience bewilderment of the illimitate multiplicity (Gergen, 1991, p. 80). The way in which affective sexual relationships are understood is subverted; thus, the conditions are fulfilled for the emergence of new forms of interrelating ourselves in an affective sexual way. Likewise, relating in a consensual non-monogamous way, or relating in a non-heterosexual way does not mean *per se* a change in the discourse and real practices that sustain and promote these new ways of relating as opposed to a whole heterosexual and monogamous socialization. Paraphrasing Kollontai (1976), we could consider that a change in the formal or external ties that unite a couple does not mean “wipe out in one act” romantic considerations—maintained by a patriarchal system—that over decades have built and validated a way of understanding the relational dynamics, promoting some at the expense of others. That is why, and this time, following the research of Lagarde (2001), that revolution is not, in an intrinsic manner, merely in the way of the relationships, and thus introducing a new person to the relationship or agreeing to maintain an open or polyamorous relationship does not entail a deconstruction of romantic considerations per se.

As Hammack et al. (2019) points out. In the 21st century, greater diversity in human relationships proliferates, and as we have noted, new relational ways and forms of intimacy emerge. As Fairbrother et al. (2019) shows, although only a small proportion of the population currently maintains a consensual non-monogamous relationship, interest in consensual non-monogamous relationships is greater, especially among younger adults, who also have a greater interest in and commitment to these types of relationships, suggesting that consensual non-monogamous relationships may increase in prevalence over time. Despite this, people in consensual non-monogamous relationships are perceived less favorably than those in monogamous relationships (Grunt-Mejer and Campbell, 2016) considering this type of relationship to be of poorer quality, even rating consensual non-monogamous people with arbitrary traits (Conley et al., 2013).

Queer studies provide a flexible paradigm that recognizes the diverse forms and possibilities in a post-normative and post-binary context in which people are not constrained by hegemonic assumptions belonging to the paradigms of gender, sexuality and relationships (Hammack et al., 2019). Furthermore, sexual and gender identities are deeply and recursively multifaceted (Van Anders, 2015), which in turn are not fixed, but are fluidly generated through recurrent social practices and adherence to normative standards (Butler, 1990). Queer paradigms understand this diversity in human relations subject to historical and cultural contingencies that reconfigure our understanding of the meanings and possibilities of human bonds (Hammack et al., 2019).

## Research on Love

Myths of romantic love are defined by Yela (2003) as a set of socially shared beliefs about the supposed true nature of love. To Ferrer et al. (2010), myths of romantic love are like any other myth: fictitious, absurd, misleading, irrational, and impossible to fulfill. Yela (2003) systematizes the main shared and socially accepted myths of romantic love: (a) myth of the better half: the couple we have chosen was predestined and was the only or best possible option; (b) myth of exclusiveness: we can only feel love for one person at the same time; (c) myth of fidelity: passionate, romantic and erotic desires must be satisfied exclusively with one's partner; (d) myth of jealousy: the belief that jealousy is an indicator of true love; (e) myth of marriage: passionate love must lead to a stable cohabitation of the couple; (f) myth of eternal passion: passionate love in the first months can and must go on forever; (g) myth of equivalence: belief that the concepts of love and falling in love are equivalent, and therefore, if you stop being in love, it means that you do not love your partner anymore; (h) myth of omnipotence: belief that “love can do everything” and must remain no matter what happens in the relationship; and (i) myth of couple: the monogamous couple is something natural and universal, and it has always been at any time and in any culture.

Research about love has mainly focused on the study of a specific type of love, romantic love, which, framed in a specific historical, social and cultural context, has been an important part of our socialization with respect to hegemonic affective sexual relationships, such as heterosexual and monogamous ones (Hendrick and Hendrick, 1986; Barcelona, 1992; Glucksberg and McGlone, 1999; Flecha et al., 2005; Lagarde, 2005; Lakoff and Johnson, 2008; Schäfer, 2008; Herrera, 2010; Cubells and Calsamiglia, 2015; Sánchez-Sicilia and Cubells, 2019).

Regarding romantic love and its relationship with sex, romantic love expresses a certain gender ideology in which roles are differentiated, responding to a binary that places “man/woman” in positions that are not only antagonistic but also unbalanced (Marín Rojas, 2015). One of its main consequences is to increase inequality between men and women, perpetuate roles and increase life dissatisfaction (Martínez-Gómez et al., 2019). In this way, several studies coincide in the thesis that this way to conceive love is an intrinsic part of women's subordination, justifying a greater tolerance for situations of violence in affective sexual relationships in which the exercise of asymmetric power relations between men and women is legitimized, supported by the process of socialization in love in which roles and expectations are attributed in different ways according to sex (Hooks, 2000; Bosch et al., 2007; Gil and Lloret, 2007; Pujal, 2007; Esteban and Távora, 2008; Cubells and Calsamiglia, 2015; De Miguel, 2015; Bonilla et al., 2017; Caro and Monreal, 2017; Sánchez-Sicilia and Cubells, 2019). In this context, the myths of romantic love play a fundamental role, as they are the main guides of the appropriate ways of feeling, thinking and behaving, favoring the standardization of heterosexual, romantic, monogamous, and hard-wearing relationships (Cubells and Calsamiglia, 2015; Sánchez-Sicilia and Cubells, 2019). Despite this, and as will be observed throughout this work, the vast majority of research on the myths of romantic love are focused on studying the relationship between myths and sex.

Different research has investigated the relationship between sex and the assumption of the myths of romantic love, delimited mainly in normative relations, such as monogamous and heterosexual relationships. There are studies like those of Rodríguez-Castro et al. (2013) and Rodríguez-Castro and Alonso-Ruido (2015) which indicate that women agree more than men with the beliefs of romantic love. In looking for more specificity in the possible differences by sex in romantic beliefs, there are several studies, both quantitative and qualitative, that have pointed out that in women romantic love and a more idealized, love-mate, and less ludic vision of love predominates, where passion is linked to altruism and sacrifice, while in men sexual and ludic love predominates, focused on an objective, where they enjoy superficial and transitory relationships (Ubillos et al., 2001; Leal, 2007; Rodríguez-Castro et al., 2013; Caro and Monreal, 2017; Sánchez-Sicilia and Cubells, 2018). On the contrary, Ferrer et al. (2010), Ramos et al. (2010), and Larrañaga et al. (2012) observe that these beliefs are present in boys and girls, without finding significant differences by sex, nor do Cuenca-Montesino et al. (2015) find them with respect to the intensity of romantic love. On the other hand, results like those of Fundación Mujeres (2011), Nava-Reyes et al. (2018) and Bisquert-Bover et al. (2019) claim that men respond in a more mythical way about love. Several studies have addressed the assumption of romantic love myths and their relationship with sex, but there is no consensus that there are differences between women and men with respect to adherence to this type of belief.

Martínez-Gómez et al. (2019) states that the results of studies on sex make many of these myths associated with heterosexual couples and relationships, making the LGTBI collective invisible. For this reason, in this work, we considered it fundamental to study the myths of romantic love and sexual orientation. Although no specific works have been found in relation to myths of romantic love and sexual orientation, we found several works that studied aspects that are included in myths of romantic love, such as those that refer to jealousy and exclusivity. Dijkstra et al. (2013) observe that lesbian women and gay men express less intensity of jealousy than heterosexual people before hypothetical scenarios of lack of exclusivity. Atencio (2017) found that bisexual people show higher levels of jealousy than homosexual and heterosexual people before a possible situation of lack of exclusivity, with the latter group experienced a lower level of jealousy and discomfort. Frederick and Fales (2016) found in their work with a sample of 2,275 bisexual people that only one third of bisexual women and men in the study had discomfort in a situation of lack of sexual exclusivity. The same authors found that in this study with a sample of 1,588 homosexual people, that in the same way as with bisexual people, only one third of the homosexual people in the study reported discomfort in a situation of lack of sexual exclusivity, and no differences were found between gay and lesbian participants in terms of the degree of discomfort.

Regarding the relationship that romantic love has with the type of affective sexual relationship practiced, some articles, especially qualitative studies, discuss a few types of non-hegemonic relationships in depth. However, few investigations delve into the possible similarities or differences between this type of relationship and traditional monogamous ones (Balzarini et al., 2019a,b). In one of the first works on the subject of study, Klesse (2006)—who interviewed non-heterosexual people in non-monogamous sexual relationships—found that polyamorous people constructed the term polyamory as a consensual non-monogamy, away from the principles of monogamy and romantic love. Later, both Wilkinson (2010) and Klesse (2011) himself, observed that at least some aspects of the discourses of romantic love had been absorbed by the notion of polyamory, evidenced by the close interrelationship between love, intimacy, affection, and sexual desire in polyamory. This seems to be consistent with the research findings of Morrison et al. (2013), who found no difference between monogamous and non-monogamous relationships with regard to passionate love, confidence and the attachment pattern. For Enciso (2015), polyamory is not the antithesis of monogamy, but the two concepts have several similarities, suggesting that it might sometimes be more accurate to refer to polyamory as “polymonogamy.” Following along this line, Ben-Ze'ev and Brunning (2018) consider that polyamory represents a romantic way of life with self-expansive criteria, whereas Wosik-Correa (2010) points out that romantic love discourses value individuality in the same way as non-monogamous discourses do; even though there is not an expectation of sexual fidelity to a single partner, there is a certain type of “emotional fidelity” toward those forming the relationship that has been reconfigured (Klesse, 2011). Thus, the studies presented suggest that consensual non-monogamous forms do not differ substantially from romantic forms and that they have also incorporated characteristics of the latter. According to the findings of Balzarini et al. (2019a) it would not be enough to compare consensual non-monogamous relationships with monogamous ones, as the different configurations of polyamorous relationships would influence the similarities and differences that might exist with monogamous relationships.

In this context, and considering the lack of consensus regarding possible sex differences in the assumption of the myths of romantic love and the scarcity of quantitative studies that investigate the assumption of these beliefs and their relationship with sexual orientation and consensual non-monogamous forms of sex-affective relationships, the present research aims to address this objective and identify the role of sex, type of sexual affective relationship, and sexual orientation in the assumption of the myths of romantic love. To address this objective, a questionnaire had to be created and its psychometric properties evaluated. Our main hypothesis is that there will be no difference in the assumption of the myths of romantic love based on sex. With respect to the sexual orientation variable, and based on the extensive literature presented in this paper that relates the discourse of romantic love as part of heterosexual socialization, as well as quantitative studies that emphasize the study of jealousy and lack of exclusivity as a function of sexual orientation, we consider that there will be significant differences depending on sexual orientation. In terms of the type of affective-sexual relationship and by referering to other qualitative studies, we anticipate that there will be no significant differences according to the type of relationship.

## Method

### Participants

Using a non-probabilistic sampling for convenience and snowball type, which was accessed through a social network system as a means of calling and selecting participants, a sample of 1,235 people who participated between April and May 2018 was accessed. Of these participants, 81.5% reported having Spanish nationality, 12% Chilean, 1.1% Mexican, and 6% another nationality (Italian, Argentine, Venezuelan, Romanian, Portuguese, among others). Regarding the level of studies completed or currently completed, 57.2% had university studies, 26.9% a master's or postgraduate degree, 10.9% higher professional education, 4.5% general secondary education, and 0.5% were other. The 68.9% (*n* = 851) stated that they were women, 30.2% (*n* = 373) men, and 0.9% (*n* = 11) stated that they did not feel represented by this dual category. The average age was 26.92 years (SD: 8.51), between a minimum range of 18 and a maximum of 70, with a median of 24 and a mode of 19. Concerning sexual orientation, 60.7% (*n* = 750) stated that they were heterosexual, 24% (*n* = 296) bisexual, 9.5% (*n* = 117) homosexual, and 5.8% (*n* = 72) stated that they did not feel represented by any of these categories (some participants identified themselves as asexual, demisexual, pansexual, undefined, “hetero-curious,” and queer, among others). Regarding the type of affective-sexual relationship, 59.4% (*n* = 734) recognized that they were maintaining or had maintained a monogamous relationship, 33.1% (*n* = 409) were maintaining or had maintained consensual non-monogamous relationships (open relationships, polyamory, relationship anarchy, or swinging), 6.1% (*n* = 76) stated that they were not maintaining or had not maintained any type of affective-sexual relationship and 1.3% (*n* = 16) stated an “unclassifiable” types of affective-sexual relationship. More details for the sample and the data can be found on the Open Science Framework (OSF; see: https://osf.io/q4gb9/).

### Instrument

#### Affective Sexual Diversity Evaluation Scale

The affective sexual diversity evaluation survey is a self-application form designed by the authors of this study for the objectives of the research, based on the categorization presented by Barrón et al. (1999), Yela (2003), and Ferrer et al. (2010) and designed with items formulated in a non-exclusive way toward non-hegemonic relationships.

The survey language was Spanish. It was composed of a descriptive section and three scales structured in the following way: (A) descriptive and sociodemographic data; (B) scale of feelings toward the relationship(s) adapted to affective sexual diversity; (C) scale of sexual and emotional jealousy adapted to affective sexual diversity and (D) scale of myths of romantic love adapted to affective sexual diversity. To respond to the objectives proposed in this article, only sections A and D were used, which are detailed below.

##### Descriptive and Sociodemographic Data (A)

This section of the instrument is made up of items that collect sociodemographic information, such as sex, age, sexual orientation, experience(s) of sexual relationship(s) current or past- in the case of not having a relationship at the moment, you were asked to answer according to the last sexual affective relationship that you had-, etc. In addition, there was an item with 17 response options corresponding to different types of affective sexual relationships- a categorization elaborated for the objectives of the research based on the initial categorization of Rohwer (2015)—which ranged from monogamy to different expressions of consensual non-monogamy: faithful monogamy, unfaithful monogamy (me), unfaithful monogamy (him/her), unfaithful monogamy (both), open relationship (emotional and sexual) of “limited” communication, open relationship (emotional and sexual) of “open” communication, open relationship (emotional but not sexual) of “limited” communication, open relationship (emotional, but not sexual) of “open” communication, open relationship (sexual but not emotional) of “limited” communication, open relationship (sexual but not emotional) of “open” communication, hierarchical closed-triad polyamory, non-hierarchical closed-triad polyamory, hierarchical open-triad polyamory, non-hierarchical open-triad polyamory and swinging. In addition, for those who did not feel represented by the alternatives we proposed, the response was considered an open option item. Each response option is accompanied by corresponding explanatory texts and drawings (Annex 1).

For the development of the analyses presented in this article, the first four response options were grouped into the “monogamous” category, and the following ones as well as those of the open option, which coincided with a type of consensual non-monogamous relationship, were grouped in the “consensual non-monogamous” category. Those who marked the option of never having had any type of affective sexual relationship were grouped in the “unrelated” category. Those responses from the open option that was unclassifiable were not considered in the analyses.

To evaluate the sexual orientation variable, participants had to choose one of the following options: heterosexual, homosexual, bisexual, other (indicate which other). Regarding the sex variable, it was understood as an identity and non-binomial category where participants choose among three options [man, woman, other (to complete)] in relation to the sex with which they identified.

##### Scale of Myths of Romantic Love Adapted to Affective Sexual Diversity (D)

The scale consists of 12 items presented in a Likert-type response format that goes from 1 “strongly disagree” to 5 “strongly agree.” The items that are inverse are presented with the letter R at the beginning of code D1. Affective sexual relationships must always be composed of two people—D2. Affective sexual relationships must be directed toward a stable and hard-wearing union—RD3. We are capable of falling in love with more than one person at a time—D4. To be jealous is an indicator of true love—D5. In the case of falling in love with two people at the same time, we will always be more in love with one than with the other; we will never feel precisely the same for both—RD6. We can love more than one person at a time—D7. In the case of loving two people at the same time, we will always love one more than the other; we will never feel the same for both—RD8. When in an affective sexual relationship, there is no problem having sexual relationships with other people—RD9. When in an affective sexual relationship, there is no problem in maintaining emotional (non-sexual) relationships with other people—D10. An affective sexual relationship must lead to a stable and forever union—RD11. You can be “complete” without having an affective sexual relationship—D12. Somewhere there are people who are predestined to be with others and start an affective sexual relationship. The global scale composed of the average of all the items is presented. A higher score on the total scale will mean a higher adherence to the myths of romantic love, while a lower score will represent a lower adherence to these beliefs.

The internal consistency for this scale (*n* = 1,235) presents a Cronbach's alpha of 0.834, which shows a reliability value that is in line with what is expected to be applied to research (Nunnally, 1978).

### Procedure

The construction of the scale of romantic love myths adapted to sexual diversity was carried out based on a bibliographical review of works that had previously studied the phenomenon of myths about romantic love, principally the categorization of Barrón et al. (1999), Yela (2003), and the study by Ferrer et al. (2010) mentioned above. A scale was designed with items formulated in a non-exclusive way toward non-hegemonic types of relationships. Traditionally, the scales of romantic love myths have been framed within the limits of heterosexual monogamy. For this purpose, the following procedure was developed:

1) Creation of the items by excluding terms that refer only to normative affective sexual relationships and making other forms of relationship invisible was undertaken.

2) Expert judgment was used to assess the content validity. Judges assessed the degree to which the instrument measures the variable it seeks to measure. They are experts both in the design of instruments and in research in social sciences, critical social psychology and affective sexual diversity.

3) A discussion group was carried out with 10 young people, who reviewed in detail the understanding and scope of each item. Their suggestions were taken into account for further revision of the instrument.

4) A pilot study was carried out with a sample of 50 people, in which information about the difficulties in understanding some items was collected. Likewise, we identified the necessity of incorporating new items. Finally, the scale was composed of 12 items.

5) Before the massive application, we requested the collaboration of an expert in the diffusion of knowledge regarding love and consensual non-monogamous relationships, with the aim of correcting those ambiguities or difficulties that the instrument could still present concerning the non-normative forms of affective sexual relationships.

Later, a massive implementation was carried out to fulfill the aims of this study. Participants from the general population were invited to participate, and the questionnaire was disseminated on a social network system, inviting all those who were interested to complete it and inspiring them to disseminate it among their contacts.

Participants who were or had been in a consensual non-monogamous affective sexual relationship were intentionally invited to participate, with the aim of having a wide sample of people who could relate in this way. To that end, the instrument was disseminated among various Facebook groups that address non-normative content regarding affective sexual relationships.

This procedure required research staff to make prior contact with those who managed these online spaces to explain the objectives of the research and propose inviting their members. Finally, the instrument was applied in the groups Poliamor Catalunya, Poliamor Chile, Golfxs con Principios, Poliamor Salamanca, Alchimia Poliamor Chile, Poliamor España, and Poliamor Valencia. Regarding the ethical safeguards, the participants gave their informed consent prior to the administration of the instrument. Before the application of the survey, the participants provided informed consent, which was created for the purposes of this research. The document considers the norms and criteria proposed by the Code of Ethics of the American Psychological Association and the Singapore Declaration, ensuring the well-being of the participants, their voluntary participation, anonymity, and confidentiality.

### Data Analysis

We first analyzed the factorial structure of the scale of myths of romantic love, for which the sample was divided into two groups. With the first subsample, exploratory factor analysis (EFA) was carried out to identify the underlying structure of the data, using principal components and Varimax rotation as a method of extraction. Straightaway, we carried out a confirmatory factor analysis (CFA) with the remaining 50% of the sample to confirm the factor structure proposed by the EFA. To estimate the goodness of fit of the model, we used chi-square (χ^2^) not significant, the Comparative Fit Index (CFI > 0.95), the RMSEA (<0.08), the Tucker-Lewis Index (TLI > 0.95), and the SRMR (<0.05) (Byrne, 2006; Ortiz and Fernández-Pera, 2017). An Ordinal Cronbach's alpha was calculated for the total scale and the two factors. The quantitative variables are described by the mean (M) and the standard deviation (SD). For qualitative variables, case counts and percentages reported.

The Student's *t*-test was used to analyze whether there are differences by sex in the scores of the different items of the romantic love myths instrument, as well as the total scale and the two factors that make up this scale (its factorial structure is presented in the results section). In addition, in order to analyze differences according to sexual orientation and the type of relationship separately, a one-way ANOVA test was used. For statistically significant variables we carried out comparisons by pairs, applying Tukey's test. However, in the case of the analyses by individual items, *post-hoc* ANOVA analyses were not reported, since the total score of the instrument, that is the total mark and the results of its component factors, were considered more relevant in this study.

It is important to note that for all analyses incorporating the sex variable, the “other” group (*n* = 11) was excluded, and when the type of relationship variable was analyzed, the “unclassifiable” group (*n* = 16) was excluded due to the very small sample sizes.

To analyze the combined effect of the variables of interest (sex, sexual orientation, and type of relationship) on the dependent variable referring to the total romantic love myths score, the results of the multifactorial ANOVA analysis are presented to determine the effect for each one of them. These analyses were also carried out incorporating each of the factors of the instrument as a dependent variable.

The significance level was set at 5% (*p* ≤ 0.05). The specific *p*-values obtained are presented in the different analyses, except when the values are <0.001, in which case they are indicated as *p* < 0.001.

In the different group comparison analyses, data referring to effect size are presented. Specifically, Cohen's d (d) was used in the case of two-group comparisons, while eta squared (η^2^) was used for ANOVA. According to Cohen (1988), the reference values for d are: <0.49 corresponds to a small effect size; between 0.50 and 0.79, medium; 0.80 or more, large. Regarding η^2^, the values proposed by Cohen (1988) are: <0.05, small; between 0.06 and 0.13, medium; 0.14 or higher, large.

The statistical analyses were carried out using the free software R version 3.5.3.

## Results

### Factorial Analysis

For EFA, we used the extraction method of principal components and Varimax rotation. The results show the existence of two factors that together explain 48.48% of the variance. The structure and factor loads are shown in Table 1.

**Table 1:** Factorial structure with EFA and the factor loads of the romantic love scale adapted to affective sexual diversity.

***N^**°**^***	**Item**	**Factor 1**	**Factor 2**
3	We are capable of falling in love with more than one person at a time.	0.786	
9	When in an affective sexual relationship, there is no problem in maintaining emotional (non-sexual) relationships with other people.	0.760	
8	When in an affective sexual relationship, there is no problem in having sexual relationships with other people.	0.752	
6	We are able to love more than one person at a time.	0.712	
1	Affective sexual relationships must be always composed of two people.	0.502	
5	In the case of falling in love with two people at the same time, we will always love one more than the other; we will never feel exactly the same for both	0.467	
7	In the case of loving two people at the same time, we will always love one more than the other; we will never feel exactly the same for both	0.455	
10	An affective sexual relationship must lead to a stable and forever union.		0.750
2	Affective sexual relationships must be directed toward a stable and hard-wearing union.		0.729
4	To be jealous is an indicator of true love.		0.640
12	Somewhere there are people who are predestined to be with others and start an affective sexual relationship.		0.567
11	You can be “complete” without having an affective sexual relationship.		0.390
	Explained variance	37.150%	11.329%

*Factor 1: exclusiveness; Factor 2: better half*.

*Source: Authors' own creation*.

Factor 1 corresponds to items referring to myths about exclusivity in the couple, while factor 2 alludes to items linked to the romantic idea of “the better half.”

**Figure 1:**
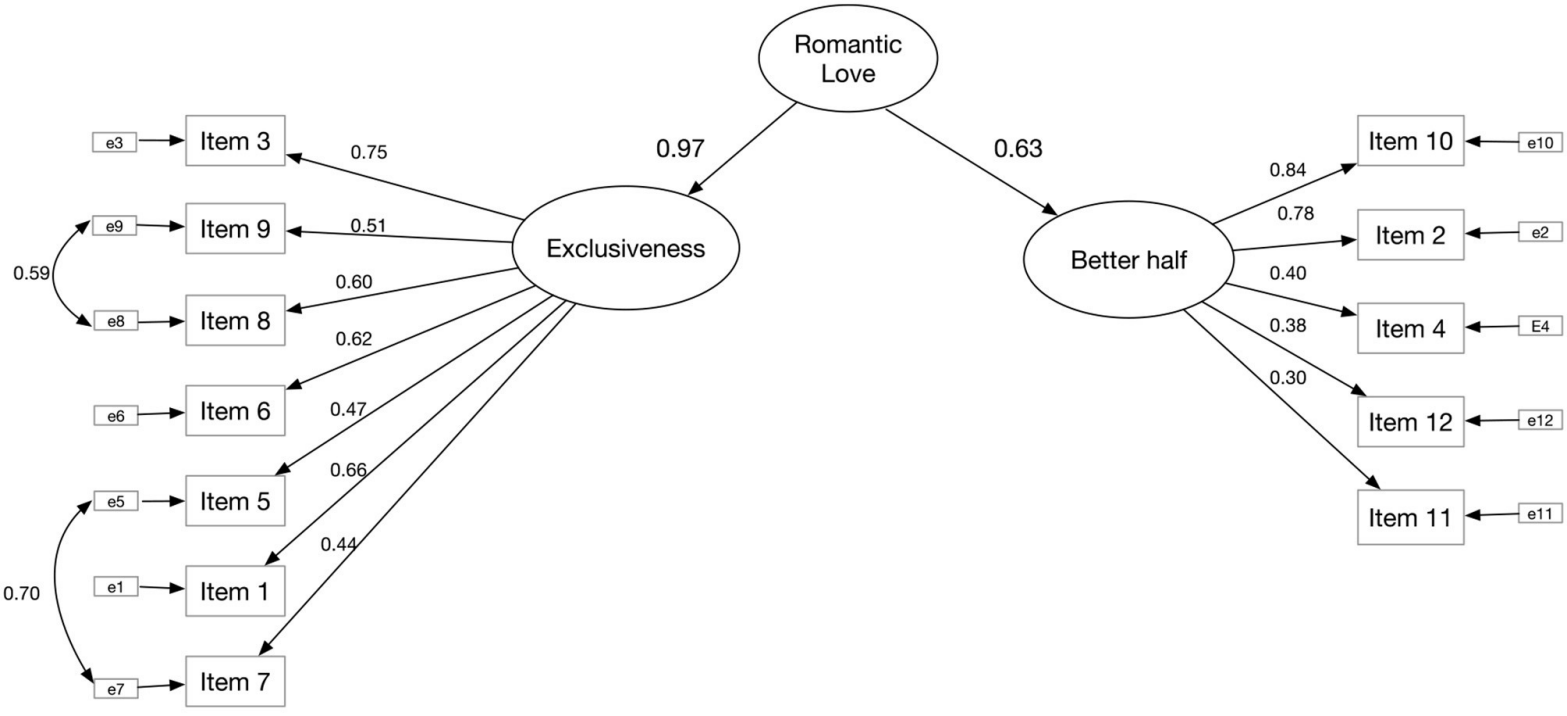
Proposed CFA structure and factor loads. *Source:* Authors' own creation.

Regarding the CFA, the Satorra-Bentler adjustment was used to carry out the analyses. The proposed structure can be seen in Figure 1. The adjustment indicators are: (χ^2^ (SB) (50) 204.70, *p* < 0.001, RMSEA (SB) = 0.070 [0.070–0.089], CFI (SB) = 0.930, TLI (SB) = 0.907, SRMR = 0.061). Two covariances were included—one between item 9 and 8 and the other between item 5 and 7—because the indicators of goodness of fit improved and because of the high correlation between the items (Table 1).

The reliability of the full scale, based on the full sample of participants, gives a Cronbach's alpha of 0.86 (CI [0.85–0.88]), while for the exclusivity factor, the reliability was 0.85 (CI [0.83–0.87]), and for the better half, it was 0.76 (CI [0.72–0.78]).

### Descriptive Analysis of the Scale of Myths of Romantic Love Adapted to Affective Sexual Diversity and Comparison of Groups

The main results obtained in the descriptive values (M and SD) for each of the items, including the total score of the myth scale and its two factors, for the variables sex (Table 2), sexual orientation (Table 3), and type of relationship (Table 4), presenting, analysis of comparison groups.

**Table 2:** Descriptive and comparative analysis, according to sex, of the Scale of Myths of Romantic Love.

	**Women**		**Men**					
	***n=*** **837**		***n=*** **371**					
	**M**	**SD**	**M**	**SD**	***t***	***p***	***d***	**C.I. 95% Cohen's d**
D1	1.92	1.24	1.96	1.21	−0.46	0.649	0.03	−0.09–0.15
D2	2.32	1.30	2.58	1.34	−3.26	0.002^*^	0.20	0.08–0.32
D3	2.10	1.21	2.04	1.20	0.85	0.398	0.05	−0.07 to 0.18
D4	1.36	0.78	1.45	0.84	−1.83	0.068	0.11	−0.01 to 0.24
D5	2.95	1.24	2.92	1.32	0.45	0.650	0.03	−0.09 to 0.15
D6	1.65	0.97	1.67	0.95	−0.31	0.758	0.02	−0.10 to 0.14
D7	2.93	1.27	2.98	1.33	−0.69	0.492	0.04	−0.08 to 0.16
D8	2.93	1.38	2.77	1.38	1.89	0.059	0.12	−0.01–0.24
D9	3.02	1.33	2.85	1.34	2.02	0.044^*^	0.13	0.01–0.25
D10	2.04	1.16	2.25	1.22	−2.88	0.004^*^	0.18	0.06–0.30
D11	1.59	1.01	1.89	1.21	−4.19	<0.001^**^	0.28	0.16–0.40
D12	2.63	1.35	2.48	1.39	1.78	0.075	0.11	−0.01 to 0.23
Myths Scale	2.29	0.71	2.32	0.74	−0.74	0.460	0.04	−0.01–0.16
Factor 1	2.50	0.85	2.46	0.88	0.85	0.394	0.01	−0.08 to 0.17
Factor 2	1.99	0.73	2.13	0.82	−2.91	0.004^*^	0.18	0.06–0.31

* *p <0.05*;

** * <0.001 Factor 1: exclusiveness; Factor 2: better half*.

*Source: Authors' own creation*.

**Table 3:** Descriptive and comparative analysis, according to sexual orientation, of the Scale of Myths of Romantic Love.

	**Heterosexual**		**Homosexual**		**Bisexual**		**Other**					
	***n=*** **742**		***n=*** **113**		***n=*** **290**		***n=*** **63**					
	**M**	**SD**	**M**	**SD**	**M**	**SD**	**M**	**SD**	***F***	***p***	***η^2^***	**C.I. 95% η^2^**
D1	2.14	1.30	1.91	1.15	1.52	0.96	1.43	0.89	22.69	<0.001^**^	0.05	0.03–0.08
D2	2.50	1.32	2.56	1.38	2.15	1.26	2.00	1.30	7.65	<0.001^**^	0.02	0.01–0.03
D3	2.34	1.23	1.99	1.20	1.61	1.00	1.48	0.93	34.25	<0.001^**^	0.08	0.05–0.11
D4	1.45	0.85	1.47	0.78	1.24	0.69	1.14	0.40	7.28	<0.001^**^	0.02	0.00–0.03
D5	3.18	1.21	2.73	1.25	2.52	1.25	2.40	1.26	26.65	<0.001^**^	0.06	0.04–0.09
D6	1.83	1.01	1.63	0.93	1.34	0.80	1.16	0.60	25.21	<0.001^**^	0.06	0.03–0.08
D7	3.13	1.23	2.99	1.31	2.57	1.30	2.33	1.33	19.14	<0.001^**^	0.05	0.02–0.07
D8	3.13	1.23	2.85	1.34	2.25	1.20	1.98	1.20	48.40	<0.001^**^	0.11	0.08–0.14
D9	3.22	1.30	3.12	1.27	2.41	1.22	2.21	1.32	35.90	<0.001^**^	0.08	0.05–0.11
D10	2.25	1.19	2.26	1.33	1.77	1.02	1.67	1.12	15.42	<0.001^**^	0.04	0.02–0.06
D11	1.81	1.15	1.74	1.24	1.43	0.91	1.24	0.62	12.36	<0.001^**^	0.03	0.01–0.05
D12	2.73	1.34	2.64	1.42	2.26	1.30	2.27	1.44	9.96	<0.001^**^	0.02	0.01–0.04
Myths Scale	2.48	0.69	2.32	0.78	1.92	0.60	1.78	0.53	61.82	<0.001^**^	0.13	0.10–0.17
Factor 1	2.72	0.83	2.46	0.89	2.03	0.71	1.85	0.65	66.10	<0.001^**^	0.14	0.11–0.18
Factor 2	2.15	0.77	2.13	0.84	1.77	0.65	1.66	0.65	24.06	<0.001^**^	0.06	0.03–0.08

** *p <0.001; Factor 1: exclusiveness; Factor 2: better half*.

*Source: Authors' own creation*.

**Table 4:** Descriptive and comparative analysis, according to type of relationship, of the Scale of Myths of Romantic Love.

	**Monogamous**		**Consensual**		**Unrelated**					
	***n=*** **734**		**Non- monogamous** ***n=*** **399**		***n=*** **75**					
	**M**	**SD**	**M**	**SD**	**M**	**SD**	***F***	***p***	***η^2^***	**C.I. 95% η^2^**
D1	2.17	1.29	1.38	0.84	2.60	1.28	73.43	<0.001^**^	0.11	0.08–0.14
D2	2.55	1.34	2.04	1.21	2.79	1.32	23.782	<0.001^**^	0.04	0.09–0.06
D3	2.37	1.22	1.46	0.84	2.56	1.37	91.98	<0.001^**^	0.13	0.10–0.17
D4	1.46	0.83	1.22	0.67	1.57	0.93	14.64	<0.001^**^	0.02	0.01–0.04
D5	3.20	1.20	2.45	1.21	3.04	1.38	48.57	<0.001^**^	0.07	0.05–0.10
D6	1.88	1.05	1.24	0.61	1.68	0.98	61.41	<0.001^**^	0.09	0.06–0.12
D7	3.19	1.23	2.46	1.23	3.11	1.32	44.827	<0.001^**^	0.07	0.04–0.10
D8	3.36	1.27	1.91	1.01	3.37	1.31	198.23	<0.001^**^	0.25	0.21–0.29
D9	3.40	1.21	2.15	1.16	3.11	1.29	140.79	<0.001^**^	0.19	0.15–0.23
D10	2.30	1.22	1.71	1.02	2.28	1.18	33.95	<0.001^**^	0.05	0.03–0.08
D11	1.74	1.11	1.56	1.04	1.71	1.02	3.64	0.027^*^	0.01	0.0–0.02
D12	2.74	1.36	2.26	1.34	2.79	1.23	17.91	<0.001^**^	0.03	0.01–0.05
Myths Scale	2.53	0.69	1.82	0.53	2.55	0.66	165.90	<0.001^**^	0.22	0.18–0.25
Factor 1	2.80	0.80	1.86	0.62	2.78	0.79	209.12	<0.001^**^	0.26	0.22–0.30
Factor 2	2.16	0.77	1.76	0.68	2.22	0.68	40.92	<0.001^**^	0.06	0.04–0.09

* *p <0.05*;

** *p <0.001; Factor 1: exclusiveness; Factor 2: better half*.

*Source: Authors' own creation*.

Regarding sex, statistically significant differences, although with a small effect size, are observed in items 2 [*t*_(1,206)_ = −3.26, *p* = 0.002, *d* = 0.20], 9 [*t*_(1,206)_ = −2.02, *p* = 0.04, *d* = 0.13], 10 [*t*_(1,206)_ = −2.88, *p* = 0.004, *d* = 0.18] and 11 [*t*_(1,206)_ = −4.19, *p* < 0.001, *d* = 0.28]. In all these items, except item 9, it is the men who present a higher score.

Despite finding some differences regarding sex in some items (Table 2), it was observed that there are no statistically significant differences between women and men on the global scale of myths [*t*_(1,206)_ = −0.74, *p* = 0.46, *d* = 0.04].

In Factor 1, as shown in Table 2, no statistically significant differences were found according to sex [*t*_(1,206)_ = 0.85, *p* = 0.39, *d* = 0.01], while in Factor 2 men had a higher score than women, although with a small effect size [*t*_(1,206)_ = −2.91, *p* = 0.004, *d* = 0.18].

Regarding sexual orientation, as can be seen in Table 3, statistically significant differences were observed in all items. Those items in which the effect size was medium are mentioned below: item 3 [*F*_(3, 1,204)_ = 34.25 *p* < 0.001, η^2^ = 0.08], item 5 [*F*_(3, 1,204)_ = 26.65 *p* < 0.001, η^2^ = 0.06], item 6 [*F*_(3, 1,204)_ = 25.21, *p* < 0.001, η^2^ = 0.06], item 8 [*F*_(3, 1,204)_ = 48.40 *p* < 0.001, η^2^ = 0.11], and item 9 [*F*_(3, 1,204)_ = 35.90, *p* < 0.001, η^2^ = 0.08].

One-way ANOVA analyses revealed significant differences for the sexual orientation variable in the global romantic love myths score [*F*_(3, 1,204)_ = 61.82 *p* < 0.001, η^2^ = 0.13] with a medium effect size (Table 3). Specifically, the heterosexual group presented higher scores with respect to the bisexual group (mean difference = 0.56, SE = 0.05, *p* < 0.001, *d* = 0.84) and the “other” category (mean difference = 0.71, SE = 0.08, *p* < 0.001, *d* = 1.03). In addition, homosexual people scored higher than bisexuals (mean difference = 0.40, SE = 0.07, *p* < 0.001, *d* = 0.61) and others (mean difference = 0.55, SE = 0.11, *p* < 0.001, *d* = 0.77).

Regarding Factor 1, statistically significant differences were also found according to sexual orientation, with a large effect size, as shown in Table 3 [*F*_(3, 1,204)_ = 66.10 *p* < 0.001, η^2^ = 0.14]. Specifically, the heterosexual group presents higher scores than the homosexual group (mean difference = 0.26, SE = 0.08, *p* = 0.006, *d* = 0.31), bisexual (mean difference = 0.69, SE = 0.06, *p* < 0.001, *d* = 0.86) and the “other” group (mean difference = 0.87, SE = 0.10, *p* < 0.001, *d* = 1.06). In addition, homosexual people had higher scores than the bisexual group (mean difference = 0.43, SE = 0.09, *p* < 0.001, *d* = 0.56) and the “other” group (mean difference = 0.61, SE = 0.13, *p* < 0.001, *d* = 0.75).

Table 3 also shows that there are differences with respect to Factor 2, according to sexual orientation, with a medium effect size [*F*_(3, 1,204)_ = 24.06 *p* < 0.001, η^2^ = 0.06], obtaining that heterosexual people present more myths than those who define themselves as bisexual (mean difference = 0.38, SE = 0.05, *p* < 0.001, *d* = 0.51) and those who ascribe to the category “other” (mean difference = 0.49, SE = 0.10, *p* < 0.001, *d* = 0.64). In addition, the homosexual group presents higher scores in this factor, with respect to the bisexual group (mean difference = 0.36, SE = 0.08, *p* < 0.001, *d* = 0.46) and to “other” (mean difference = 0.47, SE = 0.12, *p* < 0.001, *d* = 0.60).

On the other hand, as can be seen in Table 4, statistically significant differences were detected in all the items of the instrument, when comparing them according to type of relationship. Particularly, the items in which a medium effect size is observed for the comparison are: item 1 [*F*_(2, 1,205)_ = 73.43 *p* < 0.001, η^2^ = 0.11], item 3 [*F*_(2, 1,205)_ = 91. 98 *p* < 0.001, η^2^ = 0.13], item 5 [*F*_(2, 1,205)_ = 48.57 *p* < 0.001, η^2^ = 0.07], item 6 [*F*_(2, 1,205)_ = 61.41 *p* < 0.001, η^2^ = 0.09], and item 7 [*F*_(2, 1,205)_ = 44.83 *p* < 0.001, η^2^ = 0.07]. Furthermore, in items 8 [*F*_(2, 1,205)_ = 198.23 *p* < 0.001, η^2^ = 0.25] and 9 [*F*_(2, 1,205)_ = 209.12 *p* < 0.001, η^2^ = 0.26] the effect size was large.

One-way ANOVA analyses revealed significant differences between the different groups according to the type of relationship, with respect to the dependent variable referred to the total score of the romantic love myths scale [*F*_(2, 1,205)_ = 165.90 *p* < 0.001, η^2^ = 0.22] with a large effect size. Specifically, the differences are explained by the fact that the monogamous group presents higher scores than the consensual non-monogamous groups (mean difference = 0 0.71, SE = 0.04, *p* < 0.001, *d* = 1.11), as shown in Table 4. In addition, the unrelated group presents higher scores than the consensual non-monogamous group (mean difference = 0.73, SE = 0.08, *p* < 0.001, *d* = 1.32).

Regarding factor 1, according to type of relationship, statistically significant differences were found, with a large effect size [*F*_(2, 1,205)_ = 209.12 *p* < 0.001, η^2^ = 0.26). *Post-hoc* analyses showed that the monogamous group scored significantly higher than the non-monogamous group (mean difference = 0.93, SE = 0.05, *p* < 0.001, *d* = 1.27), while the unrelated group had higher scores than the non-monogamous group (mean difference = 0.92, SE = 0.09, *p* < 0.001, *d* = 1.42).

Regarding Factor 2, statistically significant differences were also observed in the scores obtained by the different groups according to type of relationship [*F*_(2, 1,205)_ = 40.92 *p* < 0.001, η^2^ = 0.06], although the effect size in this case was medium. Specifically, it was obtained that the monogamous group scored higher than the non-monogamous group (mean difference = 0.40, SE = 0.05, *p* < 0.001, *d* = 0.64). In addition, the unrelated group presented higher scores than the non-monogamous group (mean difference = 0.47, SE = 0.09, *p* < 0.001, *d* = 0.68).

### Multifactor Analysis of Variance

The results of the analysis of the myth scale according to sex, sexual orientation and type of relationship are presented below. Multifactor analysis of variance (ANOVA) revealed that there are no statistically significant differences between women and men with respect to the total scale score [*F*_(1, 1, 185)_ = 0.19, *p* = 0.660, η^2^ = <0.01], but there are differences according to sexual orientation [*F*_(3, 1,185)_ = 10.48, *p* < 0.001, η^2^ = 0.03] and type of relationship [*F*_(2, 1,185)_ = 25.66, *p* < 0.001, η^2^ = 0.04], with a small effect size in both cases. The interaction between the different factors did not reach statistical significance. Specifically, there were no differences in this factor with respect to the interaction among sex and sexual orientation [*F*_(3, 1,185)_ = 1.36, *p* = 0.255, η^2^ <0.01], sex and relationship type [*F*_(2, 1,185)_ = 0.09, *p* = 0.910, η^2^ <0.01], sexual orientation and type of relationship [*F*_(6, 1,185)_ = 1.46, *p* = 0.188, η^2^ = 0.01]; nor between sex, sexual orientation, and type of relationship [*F*_(5, 1,185)_ = 0.97, *p* = 0.436, η^2^ <0.01].

Regarding the sexual orientation variable, there are statistically significant differences between the different groups, except between bisexual and other; and between heterosexual and homosexual as shown in Table 5. This table shows that the heterosexual group scores significantly higher than the bisexual group and the “other” group, while the homosexual group also scores significantly higher than the bisexual group and the “other” category.

**Table 5:** *Post-hoc* factorial ANOVA analysis for the dependent variable of global romantic love myths score and its two factors, considering sexual orientation as a grouping variable.

**Variable**	**Contrast**	***N***	**M**	**SD**	**Mean difference**	**SE**	***p***	***d***	**C.I. 95% Cohen's d**
Myths scale	Heterosexual Homosexual	742 113	2.48 2.32	0.69 0.78	0.16	0.06	0.052	0.49	0.29–0.69
	Heterosexual Bisexual	742 290	2.48 1.92	0.69 0.60	0.56	0.04	<0.001^**^	1.04	0.83–1.24
	Heterosexual Other	742 63	2.48 1.78	0.69 0.53	0.71	0.08	<0.001^**^	1.19	0.93−1.46
	Homosexual Bisexual	113 290	2.32 1.92	0.78 0.60	0.40	0.07	<0.001^**^	0.51	0.29–07.73
	Homosexual Other	113 63	2.32 1.78	0.78 0.53	0.55	0.10	<0.001^**^	0.60	0.29–0.92
	Bisexual Other	290 63	1.92 1.78	0.60 0.53	0.15	0.09	0.316	0.17	−0.10–0.44
Factor 1	Heterosexual Homosexual	742 113	2.72 2.46	0.83 0.89	0.26	0.07	0.002*	0.31	0.11–051
	Heterosexual Bisexual	742 290	2.72 2.03	0.83 0.71	0.69	0.05	<0.001^**^	0.86	0.82–1.00
	Heterosexual Other	742 63	2.72 1.85	0.83 0.65	0.87	0.09	<0.001^**^	1.06	0.80–1.33
	Homosexual Bisexual	113 290	2.46 2.03	0.89 0.71	0.43	0.08	<0.001^**^	0.56	0.34–0.78
	Homosexual Other	113 63	2.46 1.85	0.89 0.65	0.61	0.11	<0.001^**^	0.89	0.56–1.20
	Bisexual Other	290 63	2.03 1.85	0.71 0.65	0.18	0.10	0.288	0.26	−0.02–0.53
Factor 2	Heterosexual Homosexual	742 113	2.15 2.13	0.77 0.84	0.02	0.07	0.997	0.03	−0.17–0.22
	Heterosexual Bisexual	742 290	2.15 1.77	0.77 0.65	0.38	0.05	<0.001^**^	0.51	0.38–0.65
	Heterosexual Other	742 63	2.15 1.66	0.77 0.65	0.49	0.10	<0.001^**^	0.64	0.38–0.90
	Homosexual Bisexual	113 290	2.13 1.77	0.84 0.65	0.36	0.08	<0.001^**^	0.51	0.29–0.73
	Homosexual Other	113 63	2.13 1.66	0.84 0.65	0.47	0.11	<0.001^**^	0,60	0.29–0.92
	Bisexual Other	290 63	1.77 1.66	0.65 0.65	0.11	0.10	0.720	0.17	−0.10–0.44

*Adjusted p-values: Tukey Method*;

** *p <0.001; Factor 1: exclusiveness; Factor 2: better half*.

*Source: Authors' own creation*.

Regarding the type of relationship variable, statistically significant differences were detected between the monogamous and consensual non-monogamous groups and between consensual non-monogamous and unrelated groups, as shown in Table 6. Specifically, this table shows that the monogamous group has a statistically higher score than the non-monogamous group, while the non-monogamous group scores significantly lower than the unrelated group.

**Table 6:** *Post-hoc* factorial ANOVA analysis for the dependent variable of global romantic love myths score and its two factors, considering the type of relationship as a grouping variable.

**Variable**	**Contrast**	***N***	**M**	**SD**	**Mean difference**	**SE**	***p***	***d***	**C.I. 95% Cohen's d**
Myths scale	Monogamous No monogamous	734 399	2.53 1.82	0.69 0.53	0.71	0.04	<0.001^**^	1.11	0.98–1.24
	Monogamous Unrelated	734 75	2.53 2.55	0.69 0.66	−002	0.07	0.962	0.03	−0.27–0.21
	No monogamous Unrelated	399 75	1.82 2.55	0.53 0.66	−0.73	0.08	<0.001^**^	1.32	1.06–1.58
Factor 1	Monogamous No monogamous	734 399	2.80 1.86	0.80 0.62	0.93	0.04	<0.001^**^	1.27	1.13–1.40
	Monogamous Unrelated	734 75	2.80 2.78	0.80 0.79	0.01	0.09	0.986	0.03	−0.21–0.26
	No monogamous Unrelated	399 75	1.86 2.78	0.62 0.79	−0.92	0.09	<0.001^**^	1.42	1.15–1.68
Factor 2	Monogamous No monogamous	734 399	2.16 1.76	0.77 0.68	0.40	0.05	<0.001^**^	0.54	0.42–0.66
	Monogamous Unrelated	734 75	2.16 2.22	0.77 0.68	−0.07	0.09	0.724	0.08	−1.16–0.32
	No monogamous Unrelated	399 75	1.76 2.22	0.68 0.68	−0.47	0.09	<0.001^**^	0,68	0,42–0,93

*Adjusted p-values: Tukey Method*;

** *P <0.001; Factor 1: exclusiveness; Factor 2: better half*.

*Source: Authors' own creation*.

Regarding Factor 1, the factorial ANOVA shows that there is no interaction effect among the variables sex and sexual orientation [*F*_(3, 1,185)_ = 0.55, *p* = 0.651, η^2^ <0.01], sex and type of relationship [*F*_(2, 1,185)_ = 0.02, *p* = 0.985, η^2^ <0.01], sexual orientation and type of relationship [*F*_(6, 1,185)_ = 1.89, *p* = 0.079, η^2^ = 0.01); nor among sex, sexual orientation, and type of relationship [*F*_(5, 1,185)_ = 1.05, *p* = 0.385, η^2^ = 0.01], with respect to the score obtained in this factor, but there are differences according to sexual orientation, with a small effect size [*F*_(3, 1,185)_ = 10.73, *p* < 0.001, η^2^ = 0.03] and according to type of relationship, with a medium effect size [*F*_(2, 1,185)_ = 35.80, *p* < 0.001, η^2^ = 0.06]. As for sex case, no differences were observed in this factor [*F*_(1, 1,185)_ = 0.18, *p* = 0.668, η^2^ = <0.01].

Specifically, in terms of sexual orientation, as shown in Table 5, the heterosexual group scores significantly higher than all other groups. In addition, this table shows that the homosexual group has a statistically higher score than the bisexual group and the “other” category.

Concerning the type of relationship, Table 6 shows that monogamous people have higher scores than non-monogamous people in Factor 1, as well as the latter score significantly lower than the unrelated group.

On the other hand, as for Factor 2, the factorial ANOVA indicates that the sex variable is not significant [*F*_(1, 1,185)_ = 2.21, *p* = 0.137, η^2^ = <0.01], while sexual orientation [*F*_(3, 1,185)_ = 4.34, *p* = 0.005, η^2^ = 0.01] and type of relationship [*F*_(2, 1,185)_ = 4.26, *p* = 0.014, η^2^ = 0.01] are statistically significant, although with a small effect size. No interaction effect is observed among these different variables in terms of the score obtained in Factor 2. There were no differences in this factor with respect to the interaction between sex and sexual orientation [*F*_(3, 1,185)_ = 1.84, *p* = 0.139, η^2^ = 0.01], sex and relationship type [*F*_(2, 1,185)_ = 0.21, *p* = 0.813, η^2^ <0.01], sexual orientation and relationship type [*F*_(6, 1,185)_ = 0.89, *p* = 0.504, η^2^ <0.01]; nor between sex, sexual orientation, and relationship type [*F*_(5, 1,185)_ = 0.61, *p* = 0.694, η^2^ <0.01].

Table 5 shows that the heterosexual group has a statistically higher score than the bisexual group and the “other” category in Factor 2. The same is true for the homosexual group, whose score is statistically higher than bisexuals and “other.”

On the other hand, regarding the type of relationship, Table 6 shows that the monogamous group scored significantly higher than the non-monogamous, while the unrelated group scored statistically higher than the non-monogamous group.

## Discussion

The objective of the study was to analyze the influence of sex, sexual orientation, and type of affective sexual relationship variables on the assumption of the myths of romantic love, for which it was necessary to create and test the psychometric properties of a scale of myths about romantic love.

The instrument showed suitable psychometric properties both in terms of reliability and in terms of the validity of the construct, confirmed by the factor analyses performed (CFA and EFA). The results prove the existence of two factors corresponding to Factor 1 “exclusiveness” and Factor 2 “the better half.” Convergent validity was confirmed, showing that monogamous people scored higher than non-monogamous people on the exclusivity factor. Nevertheless, further research is needed to continue investigating the psychometric properties of the instrument.

The results regarding the relationship between sex and acceptance of romantic love myths confirmed the hypothesis that there were no significant differences between men and women. These results were observed both in the score obtained on the total scale, as well as with respect to exclusiveness factor. The results presented here contradict the expectations of some studies where women show greater agreement with the myths (Rodríguez-Castro et al., 2013; Rodríguez-Castro and Alonso-Ruido, 2015). However, the results are consistent with research in which romantic beliefs were present in equal measure in both men and women (Ferrer et al., 2010; Ramos et al., 2010; Larrañaga et al., 2012). This similarity is considered expectable, as both men and women have been socialized in patriarchal contexts that strategically promote the assumption of such myths. Likewise, by establishing gender-differentiated roles in the affective and sexual ways of relating, asymmetric power relations based on gender differences are uncritically held (Cubells and Calsamiglia, 2015). However, in the better half factor, men scored significantly higher than women, but with a small effect size. Similarly, when analyzing each item separately, significant differences were observed in which men scored higher, but with small effect sizes. It would be interesting to be able to go deeper into these differences in the future.

Regarding the sexual orientation of the participants, people who identified as bisexual and/or with other types of orientations showed a lower degree of agreement with the myths of romantic love compared to heterosexuals and homosexuals. These differences were observed in the analyses both on the global scale and when comparing item by item, both with a medium effect size. Regarding the differences observed in each of the factors, a large effect size was observed for the exclusiveness factor and a medium effect size for the better half factor. These results confirmed the hypothesis that there were significant differences according to sexual orientation. A possible explanation for this difference could be due to the characteristics of the sample, in which most bisexual people reported not establishing monogamous relationships, which represents a break with the normative forms of affective sexual relationships.

Regarding the type of affective sexual relationship, people who relate in a monogamous way and those who have never maintained an affective sexual relationship, presented a greater degree of belief in or assumption of the myths of romantic love than people who relate in a consensual non-monogamous way (open relationships, polyamory, relationship anarchy, swinging, etc.). These differences were observed for the total scale with a large effect size, as well as in the item-by-item comparison with a medium effect size. These differences were also observed in the exclusiveness factor with a large effect size and the better half factor with a medium effect size. These results disconfirmed the hypothesis that there no were significant differences according to the type of relationship. Therefore, these results provide new information which contradicts—according to statistical analysis—what has been observed by Klesse (2011), Wilkinson (2010), Enciso (2015), and Ben-Ze'ev and Brunning (2018) concerning the fact that people that have engaged in consensual non-monogamous relationships and polyamory would have incorporated romantic discourses inherent in monogamy. It would be interesting to carry out mixed studies that cross both types of data, allowing us to break down the option of consensual non-monogamy into polyamory, open relationships, and so forth, enabling us to study the possible differences that may arise.

The multifactorial analyses reinforce the findings presented above. No statistically significant differences were observed between men and women with respect to assumptions about myths of romantic love, but there were significant differences with respect to sexual orientation and type of relationship. The interaction between the different factors did not reach statistical significance. As for the factors exclusiveness and better half, the interaction among the different factors did not reach statistical significance. In both, no significant differences were observed by sex, but significant differences were observed with respect to sexual orientation and type of relationship. Thus, heterosexuals adhered most to the myths of romantic love in both the exclusiveness and better half factors, followed by homosexuals, results in line with the work of Dijkstra et al. (2013), who in turn adhered more than bisexuals and “others,” contrary to what was found by Atencio (2017) and in line with what was observed by Frederick and Fales (2016) regarding lack of exclusivity. Regarding the type of relationship, monogamous people adhered more than non-monogamous people to the myths in both the exclusivity and better half factor, while the latter adhered less than the unrelated. In both factors, the significant differences reported small effect size.

It is important to note that the effect sizes observed in the multifactor Anova analyses were smaller than those found in the one-factor Anova analyses. This could present a possible limitation to the study since, although the sample size was large, it is difficult to achieve large and similar sample sizes for all possible combinations. The groups are also very unbalanced in some cases. For example, there was a large difference in sample size between heterosexual people (*n* = 750), and those who did not feel represented by any of the proposed categories (*n* = 72). Likewise, convenience sampling may have influenced the results obtained since participants may have certain perceptions regarding the study topic (Price and Murnan, 2004). This methodological decision was taken in response to the need to have a wide sample, including a collective of difficult access, such as people who have consensual non-monogamous affective sexual relationships (*n* = 409). Likewise, it is considered necessary to make visible the low participation of men compared to women (68.9% women), both in this study and in others that deal with similar topics (Rodríguez-Castro et al., 2013; Borrajo et al., 2015). This sex difference in the number of participants could entail biases in this and other analyses.

In the future, we propose carrying out new research on the myths of romantic love, using this or other instruments with similar characteristics, with the aim that any person, regardless of how they relate to others in an affective sexual way, can feel represented, overcoming terminology such as “couple” or approaches to the myths surrounding this idea, since conceiving of affective sexual relationships as something exclusive to two people presupposes that the researcher assumes the myth of a couple (Yela, 2003). Taking into account that participants from several countries responded to the survey, it would also be of interest to develop future studies to identify possible cultural differences in the expression of the myths of romantic love, as well as possible similarities and differences in adherence to romantic love myths among the different types of consensual non-monogamous relationships as well as their contrast with monogamous couples.

Regarding the results, it is evident that the study has developed an instrument with suitable psychometric properties to evaluate the myths of romantic love in people with diverse sexual orientations and to consider the existing variety of affective sexual relationships. Moreover, the degree of assumption of romantic myths varies according to sexual orientation and type of affective sexual relationship, and not according to sex. Finally, the results report that a person, man or woman, heterosexual or homosexual, who relates in a monogamous way or who has never maintained an affective sexual relationship, will present a greater belief in or assumption of the myths of romantic love than a bisexual person or someone with another non-homosexual or heterosexual sexual orientation who has maintained at least one affective sexual relationship and who relates in a consensual non-monogamous way (open relationships, polyamory, relationship anarchy, swinging, etc.); thus, this person presents a lower assumption of the myths of romantic love.

## Data Availability Statement

More details for the sample and the data can be found on the Open Science Framework (OSF; see: https://osf.io/q4gb9/).

## Ethics Statement

Prior to the application of the survey, the participants accepted an informed consent, which was created by the researchers for the purposes of this research. The document considers the norms and criteria proposed by the Code of Ethics of the American Psychological Association and the Singapore Declaration, ensuring the well-being of the participants, their voluntary participation, anonymity and confidentiality.

## Author Contributions

JC-S, AS-S, and PA-M designed the study and acquired data, interpreted the results, and wrote the article. PA-M, AS-S, NE-N, and MB-R conducted the statistical analyses. All authors contributed to the article and approved the submitted version.

## Conflict of Interest

The authors declare that the research was conducted in the absence of any commercial or financial relationships that could be construed as a potential conflict of interest.
