# Supplementary material for: Assumption of the Myths of Romantic Love: Its Relationship With Sex, Type of Sex-Affective Relationship, and Sexual Orientation
Source: Front Sociol. 2021 May 20;6:621646. doi: 10.3389/fsoc.2021.621646 (PMC8175080; doi:10.3389/fsoc.2021.621646)
Supplement: Supplementary file 1 [file Table_1.docx]

**Annex 1**

| 1 | **Faithful monogamy**: The relationship is made up of my partner and I, we have agreed to be faithful to each other, and this is fulfilled, or I suppose it is. | 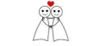 |
| --- | --- | --- |
| 2 | **Unfaithful monogamy (me)**: My partner and I make up the relationship, we have agreed to be faithful to each other, but I am (have been) unfaithful, maintaining emotional and/or sexual relationships with other people. | 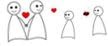 |
| 3 | **Unfaithful monogamy (him/her)**: My partner and I make up the relationship, we have agreed to be faithful to each other, but he/she is (has been) unfaithful, maintaining emotional and/or sexual relationships with other people. | 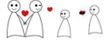 |
| 4 | **Unfaithful monogamy (both)**: My partner and I make up the relationship, we have agreed to be faithful to each other, but we are both (have been) unfaithful in maintaining emotional and/or sexual relationships with other people | 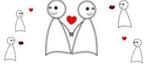 |
| 5 | **Open relationship (emotional and sexual) of "limited" communication**. The relationship is made up of another person and me. We have agreed (explicitly or implicitly) that outside of the relationship, he/she and I can have emotional and sexual relationships with others, but we prefer not to talk about what we do or do not do with those others. | 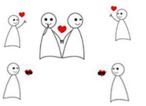 |
| 6 | **Open relationship (emotional and sexual) of "open" communication:** The same as in "5", but we prefer to talk about what we do or do not do with others. | 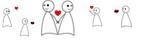 |
| 7 | **Open relationship (emotional but not sexual) of "limited" communication**: The relationship is made up of another person and me. We have agreed (explicitly or implicitly) that outside the relationship, he/she and I can have emotional (but not sexual) relationships with others, but we prefer not to talk about what we do or don't do with those others. | 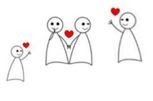 |
| 8 | **Open relationship (emotional, but not sexual) of "open" communication**: The same as in "7", but we prefer to talk about what we do or don't do with others. | 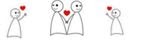 |
| 9 | **Open relationship (sexual but not emotional) of "limited" communication**: The relationship is made up of another person and me. We have agreed (explicitly or implicitly) that outside the relationship, he/she and I can have sexual (but not emotional) relationships with others but we prefer not to talk about what we do or do not do with those others. | 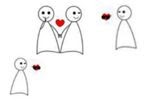 |
| 10 | **Open relationship (sexual but not emotional) of "open" communication**: The same as in "9", but we prefer to talk about what we do or don't do with others. | 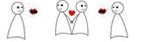 |
| 11 | **Hierarchical closed-triad polyamory**: The relationship is composed of three people, where there is a couple or a main person, who always goes first, (no necessarily there has to be an affective sexual relationship between all of them) we are faithful within the triad, we do not maintain emotional or sexual relationships outside of it, with others. | 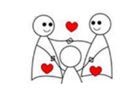 |
| 12 | **Non-hierarchical closed-triad polyamory**: The same as in "11", but generally, there are no main partners, nor is anyone more important than anyone else in terms of sexual affection. | 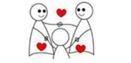 |
| 13 | **Hierarchical open-triad polyamory**: The relationship is composed of three people, where one partner is the main one and always goes first, (there does not necessarily have to be an affective sexual relationship between all of them), we can maintain or we maintain emotional or sexual relationships outside the triad, with others. | 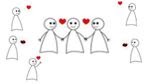 |
| 14 | **Non-hierarchical open-triad polyamory**: The same as in "13", but in general, there are no main partners, nor is anyone more important than anyone else in terms of sexual affection. | 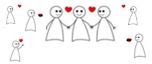 |
| 15 | **Participant swinging**: The relationship is composed by another person and me. We go together to the swinger meetings, and we maintain sexual relations with other people. | 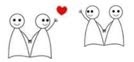 |
| 16 | **Watchful swinging**: Same as "15", but we just watch or be watched, without practicing sex with other people. | 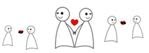 |
| 17 | Other (please write it) |  |

*Source:* Categorization elaborated by the authors based on the initial categorization of Kristin Rohwer (2015).
